# Detecting swine influenza A virus during fattening and at slaughter: implications for monitoring strategies in fattening herds

**DOI:** 10.1186/s40813-026-00529-x

**Published:** 2026-08-19

**Authors:** Pauline Deffner, Katrin Jankowitsch, Matthias Eddicks, Susanne Zöls, Mathias Ritzmann, Yury Zablotski, Robert Fux, Timm Harder, Kathrin Lillie – Jaschniski, Julia Stadler

**Affiliations:** 1 Clinic for Swine, Centre for Clinical Veterinary Medicine, Ludwig- Maximilians-Universität München, Oberschleißheim, Germany; 2 Division of Virology, Institute for Infectious Diseases and Zoonoses, Department of Veterinary Science, Ludwig-Maximilians-Universität München, Oberschleißheim, Germany; 3 Institute of Diagnostic Virology, Friedrich-Loeffler-Institut, Greifswald-Insel Riems, Germany; 4 CEVA Tiergesundheit, Düsseldorf, Germany

**Keywords:** Swine Influenza A virus, Slaughter, Monitoring, Sampling specimen

## Abstract

**Background:**

Swine influenza A virus (swIAV) is a major contributor to respiratory disease in pigs and represents a One-Health concern. This study evaluated whether slaughterhouse sampling can complement or partially substitute on-farm monitoring by comparing slaughterhouse-derived sample specimens for swIAV detection and subtype characterization.

**Results:**

Twenty-one pig farms in Germany were enrolled, and one batch of fatteners per farm was monitored longitudinally using pen-based oral fluids (OFs) at three predefined time points during fattening. In case of acute respiratory distress, tracheobronchial swabs (TBS) were collected from 15 affected pigs. At slaughter, 30 pigs per farm were sampled and tested for swIAV by qPCR, yielding OFs, nasal swabs before and after scalding (NS I/NS II), bronchial swabs (BS), lung tissue (LT), and serum. Overall, 18/21 farms (85.7%) were classified as swIAV-positive, with a seroprevalence of 92.4% in the study population. During fattening, swIAV-RNA was detected in OFs collected on-farm in 7/21 farms (33.3%) at least once, most frequently at the beginning of fattening. Among 15 farms with TBS sampling, swIAV-RNA was detected in 4 farms (26.7%). At slaughter, swIAV-RNA was detected in at least one matrix on 7/21 farms and in 74/540 pigs (13.7%). Detection probability at slaughter differed by specimen: lower respiratory tract samples showed higher detection rates and lower Ct-values than NS (BS: 9.7%, LT: 10.9%; NS: 6.3%). Diagnostic agreement between materials ranged from fair to moderate, highest between BS and LT (κ = 0.58; *p* < 0.001). Slaughterhouse OFs showed low sensitivity. From selected RT-qPCR-positive samples with Ct < 33, BS yielded the highest proportion of successfully subtyped samples. Subtypes HA-1 C.2.1 (H1avN1EA), HA-1 C.2.4 (H1avN2G), and HA-1B.1 (H1huN2G) were identified in 4/7 RT-qPCR-positive farms.

**Conclusion:**

Despite longitudinal OF sampling during fattening and additional TBS collection during acute respiratory disease, swIAV-RNA was only detected in a subset of seropositive farms. Molecular detection at slaughter was likewise restricted, whereas serology substantially improved herd-level identification. Among PCR-based sample types in slaughter pigs, BS demonstrated the highest diagnostic yield and suitability for subtype characterization. Even combined, on-farm and slaughterhouse RT-qPCR approaches remained constrained by the transient nature of swIAV shedding. Slaughterhouse sampling therefore complements—but does not replace—structured on-farm investigations for comprehensive surveillance.

## Background

Swine influenza A virus (swIAV) represents an important cause of respiratory disease in pigs worldwide, plays a major role in the porcine respiratory disease complex (PRDC) and can cause reproductive disorders in sows, negatively affecting animal health and causing considerable economic losses in pig production [1–5].

Beyond its veterinary and economic importance, swIAV is of particular concern from a One Health perspective [6, 7]. Influenza A viruses are characterized by a pronounced ability to overcome species barriers and infect a wider range of hosts [8]. Pigs can act as mixing vessels, as they can harbor multiple co-circulating IAV lineages of swine, human and avian origin, thereby facilitating viral genetic reassortment that may lead to novel virus variants with zoonotic or even pandemic potential [9–12].

In recent years, the dynamics of swIAV infections in European pig herds have changed, shifting from short, acute, and self-limiting epizootic cycles to increasingly persistent, enzootic infection patterns at farm level, often without pronounced or specific clinical signs [13–16]. These enzootic infections patterns frequently remain undetected by passive monitoring and are often characterized by low within-herd incidence, mild or subclinical clinical presentation, and recurrent infection dynamics. Importantly, coinfections with other respiratory pathogens, as part of the porcine respiratory disease complex (PRDC) play a key role in shaping the clinical manifestation and might trigger or amplify clinical respiratory disease, as interactions between pathogens can enhance disease severity and promote the emergence of overt clinical signs [3, 4]. Accordingly, several studies reported a limited or inconsistent association between clinical respiratory signs and the detection of swIAV [17, 18], emphasizing the need for active and systematic monitoring strategies. In addition to these diagnostic limitations, swIAV epidemiology at herd level is inherently complex. Field studies have demonstrated heterogeneous virus circulation, including the presence of multiple co-circulating subtypes and lineages within herds, age-dependent dynamics, and variation in subtype detection across different age groups and sampling materials [12, 13, 17, 19, 20]. Consequently, comprehensive characterization of infection dynamics and strain diversity requires multi-level sampling strategies encompassing different age groups and sample types, thereby substantially increasing the logistical demands of on-farm monitoring [17, 18]. Moreover, direct virus detection is restricted to a narrow time frame of around 7 days after infection [21–23], necessitating repeated sampling to reliably capture active infection events.

In this context, slaughterhouse-based sampling may offer a practical complementary approach for monitoring swIAV circulation in pigs, as large numbers of animals can be sampled at one single location within a short period of time, rendering the approach time- and cost-efficient. In addition, individual-animal sampling approaches such as blood collection or nasal and tracheobronchial swabs can be performed without the need for restraining live pigs, reducing handling-related stress and providing an animal welfare advantage. Furthermore, slaughterhouse sampling allows access to postmortem tissues, which may improve diagnostic sensitivity compared to sampling live animals. Data on swIAV monitoring in slaughter pigs are scarce. Available data from Europe is largely serology-based [24–26]. Research on swIAV-RNA detection at slaughter is mostly limited to Asian studies: a long‑term surveillance study in China published in 2024 found 1.95% swIAV‑RNA–positive nasal swabs in slaughter pigs, while a recent study from Laos published in 2026 reported a similar rate of 1.4% [27, 28]. While a range of sampling strategies beyond conventional nasal swabs, including environmental and group-based approaches as well as alternative individual-animal samples, has been evaluated for swIAV monitoring in suckling pigs and weaners [18, 29–31], comparable systematic evaluations on fattening farms and at the slaughterhouse level are still lacking. Against this background, the present study aimed to evaluate whether slaughterhouse sampling can support or partially substitute on-farm monitoring by comparing on-farm and slaughterhouse-derived sample matrices for swIAV detection and subtyping.

## Methods

### Study population and farm selection

The study was carried out between October 2023 and April 2024 including 21 farms located in Southern Germany. Farms fulfilling at least one of the following criteria were enrolled: history of recurrent respiratory disease, previous detection of swIAV in fattening pigs, and/or increased occurrence of lung lesions at the slaughterhouse within the preceding six months. Only farms without vaccination against swIAV in rearing and fattening animals were eligible for inclusion. The participation in the study was voluntary. The study was approved by the Ethics Committee of the Veterinary Faculty of Ludwig-Maximilians-University Munich under reference number 366-26-07-2023.

### Study design

The investigation was designed as an exploratory study conducted at 21 farms and six commercial slaughterhouses. From each farm, one batch of fatteners was sampled longitudinally during the fattening period and at slaughter. On farm cotton ropes were used to obtain oral fluid samples (OFs) at three time points (begin, mid, end of fattening). In case of respiratory signs tracheobronchial swabs (TBS) were collected within three days from 15 clinically affected pigs as part of routine diagnostic procedures. To ensure comparability across farms, pigs from the intermediate slaughter group of each batch were selected for slaughterhouse sampling procedures. Upon arrival at the slaughterhouse (SH), this group was sampled in the lairage pens via OFs. In addition, 30 pigs from each farm were randomly selected at the slaughter line after stunning and sampled individually at predefined stages of the slaughter process. Individual samples comprised nasal swabs (NS), bronchial swabs (BS), lung tissue (LT), and blood samples for serum collection. OF, NS, BS, and LT were analyzed by real-time RT-PCR for swIAV-RNA. Additionally, serum samples were examined for swIAV-specific IgG-antibodies. Sampling of 30 pigs per farm allowed the detection of a swIAV-RNA prevalence of 10% with a confidence level of 95%.

### Sample collection

#### On-farm sampling

OF sampling was performed three times during fattening: one week after entering the fattening unit (begin), between 5th and 13th week of fattening (mid), and one to three weeks before slaughter (end). OF sampling was performed using the Oral fluid Sample Collection Accessory Kit (IDEXX; USA) as described previously [32]. Briefly, three ropes per batch were placed in the pens in the fattening farms (one rope for a maximum of 25 animals) and remained there for 25 to 30 min. Subsequently, the lower, wet part of the rope was transferred into the supplied plastic bag and manually squeezed to extract the OF. The OFs were decanted into supplied 5 ml tubes and mixed with stabilizing media (Sigma-Virocult^®^ MW951S, MWE, Corsham, England) at a 1:1 ratio. For TBS collection, sterile plastic catheters (DCT catheters, Servoprax GmbH, Wesel, Germany; CH12–CH16 depending on animal size) were introduced orally after fixation with an upper‑jaw snare and the use of a mouth gag. Sampling followed the procedure described by Fablet, Marois-Créhan [33]. Catheter tips were placed in 2 mL PBS (Roti^®^‑CELL PBS, Carl Roth GmbH + Co. KG, Karlsruhe, Germany). Both OFs and TBS were subsequently stored at − 80 °C until laboratory examination.

#### Sampling at slaughter

**Fig. 1:**
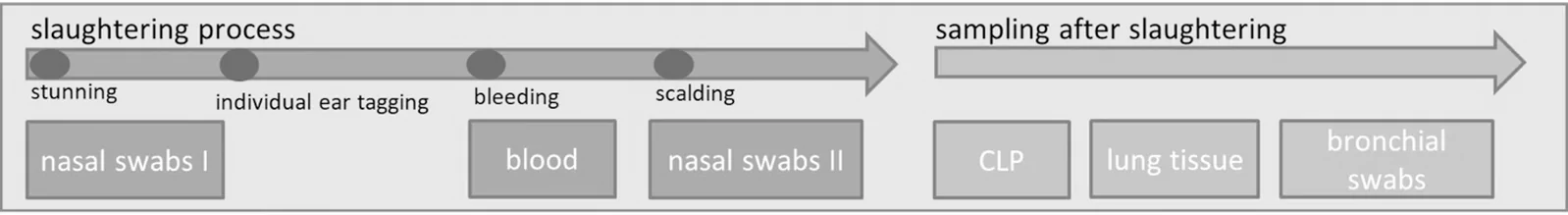
Overview of the sampling procedure during and after the slaughter process

OF sampling in the waiting area of SH was performed as described above. Further sampling procedure at slaughter is shown in Fig. 1. NS I, blood samples, and a second nasal swab (NS II) were collected during the slaughter process. After collecting NS I and blood samples, animals were individually ear-tagged to ensure accurate identification during subsequent stages of the slaughter process. NS II sampling was performed after scalding, which was conducted using scalding tanks in four SHs and spray systems in two SHs. After collection of NS I, blood samples, and NS II, the lungs of the selected animals were removed from the slaughter line and transported to the clinic for swine for further sampling (BS, LT).

Nasal swabs were obtained at two time points during the slaughter process using rayon swabs (Dryswab™ MW113, Check Diagnostics GmbH, Bad Oldesloe, Germany). The first nasal swabs (NS I) were collected immediately after stunning, the second ones (NS II) after scalding. Both nostrils were sampled by inserting the swab 2–4 cm and rotating it approximately 360 degrees. The tip of the nasal swab (3–4 cm) was placed into a plastic tube containing 1 ml of viral media (Sigma-Virocult^®^ MW951S, MWE, Corsham, England) and stored at -80 °C until laboratory analysis.

Blood samples were collected from stunned animals during exsanguination using Primavette^®^ V Serum containers (KABE Labortechnik GmbH; Germany). Blood samples were allowed to clot and centrifuged on the day of collection at 1560 g for 10 min. Serum obtained was stored at -20 °C until laboratory examination.

For bronchial swab sampling, the trachea of the lungs was opened up to the tracheal bifurcation using scissors, and subsequently, sampling was performed by swabbing (Dryswab™ MW113 from Check Diagnostics GmbH, Bad Oldesloe, Germany) both main bronchi by rotating movements as previously described [34]. The tip of the swab (3–4 cm) was placed into a plastic tube containing 1 ml of viral media (Sigma-Virocult^®^ MW951S, MWE, Corsham, England) and stored at -80 °C until laboratory examination.

Lung tissue samples were consistently taken from the right main lung lobe. As previously described by Albl, Haesner [35], a 1 × 1 cm tissue specimen was excised from the dorsal part of the right main lobe. All samples were transferred to plastic tubes (pathology tube, 20 ml, KABE Labortechnik GmbH, Nümbrecht, Germany) and stored immediately at -80 °C until further examination.

### Laboratory diagnostics

Serum samples were analyzed for the presence of antibodies to swIAV using the ID Screen^®^ Influenza A Nucleoprotein swine indirect ELISA (ID.vet Innovative Diagnostics; France). Samples were considered positive if the calculated S/P ratio was > 0.4.

Detection of swIAV-RNA in OFs, TBS, NS, BS, and LT was performed using a modified RT-qPCR targeting a fragment of the M-gene as described by Spackman, Senne [36]. Analyses were performed using an AriaMx Real‑Time PCR System (Agilent Technologies, Santa Clara, USA). OFs were analysed in pools of three, whereas NS, BS, and LT were processed in pools of five. TBS were analyzed in two pools comprising seven and eight individual samples, respectively. The examination in pools is based on previous swIAV studies, in which comparable pooling ratios did not impair RT-PCR sensitivity in either individual samples or group sample matrices [30, 37]. Viral RNA was extracted from OFs and swab samples using the High Pure Viral Nucleic Acid Kit (Roche, Mannheim, Germany) and from lung tissues using the RNeasy^®^ Mini Kit (QIAGEN GmbH, Hilden, Germany) according to the manufacturer’s instructions. RT-qPCR was performed using Master Mix QuantiTect Probe RT-PCR Kit (QIAGEN GmbH, Hilden, Germany). Individual samples from positive pools were subsequently tested separately, except for TBS samples. Samples with Ct-values ≥ 40 were considered negative. Selected positive samples with a Ct value < 33 were subjected to further subtyping using multiplex RT-qPCR assays as described by Graaf-Rau, Hennig [38].

### Statistical analysis

Statistical calculations were performed using statistical software IBM SPSS Statistics version 29 for Microsoft^®^ Windows and R version 4.5.0 (R Foundation for Statistical Computing, Wien, Österreich). The significance level was set at 0.05. A farm was considered positive if at least one of the samples investigated, regardless of the sampling material, was tested positive by RT-qPCR or ELISA. For the statistical analysis, numeric variables were tested for normal distribution. Cochran Q test was used to evaluate differences in the proportion of positive results between the different sampling materials. The probability and differences in detection odds between sampling materials for RNA- and antibody-detection were evaluated using multivariable logistic regression, with odds ratios (OR) and 95% confidence intervals reported. For quantitative RT-qPCR results, Ct-values were compared between sample materials using mixed-effects linear regression. An additional multivariable logistic regression model was used to estimate the odds of obtaining Ct-values < 33 (a proxy for subtyping suitability) by sample material, reporting ORs with 95% confidence intervals and p-values. Agreement between sampling materials (NS I, NS II, BS, LT) for swIAV-RNA detection (and separately for Ct < 33) was quantified using Cohen’s kappa (κ) with corresponding p-values. The level of agreement was calculated using Cohen’s kappa coefficient (κ). Agreement was considered poor if κ ≤ 0.2, fair if 0.21 ≤ κ ≤ 0.4, moderate if 0.41 ≤ κ ≤ 0.6, substantial if 0.61 ≤ κ ≤ 0.8, and good if κ > 0.8 [39].

All regressions and agreement analyses were restricted to individual-level sample materials (NS I, NS II, BS, LT, and serum). TBS and pen-based oral fluid samples were therefore excluded from regressions and agreement analyses, as oral fluids constitute group-level aggregates and TBS-positive pools were not subsequently resolved to individual samples, precluding animal-level comparison.

## Results

Twenty-one farms were included in the study. SwIAV exposure, as determined by serology and/or RT-qPCR, was identified in 18 of 21 farms (85.7%) (Table 1).

**Table 1:** Farm-level distribution of swIAV antibody- and RNA-positive by sample material

Farm	On - farm		Slaughterhouse					
	RT-qPCR		ELISA	RT-qPCR				
	OF	TBS pools	Serum	OF	NS I	NS II	BS	LT
1	0/3 (0%) 0/3 (0%) 0/3 (0%)	0/2 (0%)	**30/30 (100%)**	0/3 (0%)	0/30 (0%)	0/30 (0%)	0/30 (0%)	0/30 (0%)
2	0/3 (0%) **3/3 (100%)** 0/3 (0%)	**2/2 (100%) [M]**	**30/30 (100%)**	0/3 (0%)	0/30 (0%)	0/30 (0%)	0/30 (0%)	0/30 (0%)
5	**2/3 (66.7%)** 0/3 (0%) 0/3 (0%)	0/2 (0%)	**21/30 (70%)**	**1/3 (33.3%)**	**3/30 (10%)**	**2/30 (6.7%)**	**4/30 (13.3%)**	**10/30 (33.3%)**
6	0/3 (0%) 0/3 (0%) 0/3 (0%)		**30/30 (100%)**	0/3 (0%)	0/30 (0%)	0/30 (0%)	0/30 (0%)	0/30 (0%)
7	**3/3 (100%)** 0/3 (0%) **3/3 (100%)**	0/2 (0%)	**25/30 (83.3%)**	**3/3 (100%)**	**21/30 (70%)**	**30/30 (100%)**	**30/30 (100%)**	**29/30 (96.7%)**
8	0/3 (0%) 0/3 (0%) 0/3 (0%)	**2/2 (100%) [A]**	**30/30 (100%)**	0/3 (0%)	0/30 (0%)	0/30 (0%)	0/30 (0%)	0/30 (0%)
9	0/3 (0%) 0/3 (0%) 0/3 (0%)	0/2 (0%)	**30/30 (100%)**	0/3 (0%)	0/30 (0%)	0/30 (0%)	0/30 (0%)	0/30 (0%)
10	0/3 (0%) 0/3 (0%) 0/3 (0%)	0/2 (0%)	**29/30 (96.7%)**	0/3 (0%)	0/30 (0%)	0/30 (0%)	0% (0/27)	0% (0/27)
11	**3/3 (100%)** 0/3 (0%) 0/3 (0%)	0/2 (0%)	**30/30 (100%)**	0/3 (0%)	0/30 (0%)	0/30 (0%)	**3.3% (1/30)**	0/30 (0%)
12	0/3 (0%) 0/3 (0%) **3/3 (100%)**	**2/2 (100%) [E]**	**30/30 (100%)**	0/3 (0%)	**3/30 (10%)**	**2/30 (6.7%)**	0/30 (0%)	0/30 (0%)
14	0/3 (0%) 0/3 (0%) 0/3 (0%)	0/2 (0%)	**18/30 (60%)**	0/3 (0%)	0/30 (0%)	0/30 (0%)	0/30 (0%)	0/30 (0%)
15	0/3 (0%) 0/3 (0%) 0/3 (0%)		**25/30 (83.3%)**	0/3 (0%)	0/30 (0%)	0/30 (0%)	**17/30 (56.7%)**	**19/30 (63.3%)**
16	0/3 (0%) 0/3 (0%) 0/3 (0%)	0/2 (0%)	**21/30 (70%)**	**1/3 (33.3%)**	**6/30 (20%)**	0/30 (0%)	0% (0/27)	0% (0/27)
17	0/3 (0%) 0/3 (0%) 0/3 (0%)	**1/2 (50%) [A]**	**30/30 (100%)**	0/3 (0%)	0/30 (0%)	0/30 (0%)	0/30 (0%)	0/30 (0%)
18	**3/3 (100%)** 0/3 (0%) 0/3 (0%)	0/2 (0%)	**30/30 (100%)**	0/3 (0%)	0/30 (0%)	0/30 (0%)	0/30 (0%)	0/30 (0%)
20	0/3 (0%) 0/3 (0%) 0/3 (0%)		**30/30 (100%)**	0/3 (0%)	0/30 (0%)	0/30 (0%)	0/30 (0%)	0/30 (0%)
21	0/3 (0%) **3/3 (100%)** 0/3 (0%)		**30/30 (100%)**	0/3 (0%)	**1/30 (3.3%)**	0/30 (0%)	0/30 (0%)	0/30 (0%)
22	0/3 (0%) 0/3 (0%) 0/3 (0%)	0/2 (0%)	**30/30 (100%)**	0/3 (0%)	0/30 (0%)	0/30 (0%)	0/30 (0%)	0/30 (0%)
3	0/3 (0%) 0/3 (0%) 0/3 (0%)		0/30 (0%)	0/3 (0%)	0/30 (0%)	0/30 (0%)	0/30 (0%)	0/30 (0%)
4	0/3 (0%) 0/3 (0%) 0/3 (0%)		0/30 (0%)	0/3 (0%)	0/30 (0%)	0/30 (0%)	0/30 (0%)	0/30 (0%)
13	0/3 (0%) 0/3 (0%) 0/3 (0%)	0/2 (0%)	0/30 (0%)	0/3 (0%)	0/30 (0%)	0/30 (0%)	0/30 (0%)	0/30 (0%)
∑	24/189 (12.7%)	8/30 (26.7%)	499/630 (79.2%)	5/63 (7.9%)	34/630 (6.3%)	34/630 (6.3%)	52/630 (9.7%)	58/630 (10.9%)

Legend: OF: oral fluids; TBS: tracheobronchial swab; NS: nasal swab; BS: bronchial swab; LT: lung tissue; Capital letters indicate at which sampling time point during fattening swIAV-RNA was detected (B: begin; M: mid; E: end). Empty cells represent TBS samples that were not collected because no clinical respiratory signs were observed on these farms during the study period

During fattening 189 pen-based OFs were collected. In addition, 225 TBS (30 pools) were obtained from 15 farms experiencing acute respiratory signs. SwIAV-RNA was detected in OFs in 7 of 21 farms (33.3%) at least once during the fattening period. Detection occurred most frequently at the beginning of fattening (4 farms), whereas 2 farms were positive during the mid-phase and 2 farms at the end of fattening. In one farm, viral RNA was detected at more than one sampling time point. Ct-values of RT-qPCR–positive on-farm OF samples ranged from 22.6 to 35.4 (median: 26.9; IQR: 7.01). Among the 15 farms with acute respiratory signs, swIAV‑RNA was detected in four farms (26.7%). Two of these farms were sampled at the beginning of the fattening period, and one each during the mid-phase and at the end. The mean Ct-value in TBS was 23.47 (19.76–26.26; SD 2.4). In two farms (farm 2 and 12) TBS positivity coincided with OF positivity.

At slaughter 63 pen-based OFs and 3138 individual samples (NS I, NS II, Serum, BS, LT) were collected from 630 pigs. Due to severe pleuritis, complete lung retrieval at the slaughter line was not possible for all preselected animals; consequently, lung tissue samples and bronchial swabs were unavailable for six pigs from two farms. At slaughter, swIAV-RNA was detected in at least one sampling matrix in 7 of 21 farms (33.3%).

For subsequent assessment of diagnostic performance of the different sample specimens, animal‑based analyses were conducted only in the 18 farms with documented swIAV exposure.

### Detection of swIAV in different specimens at slaughter

Within the 18 swIAV-seropositive farms, swIAV-specific antibodies were detected in 92.4% (499/540) of individual serum samples. SwIAV-RNA was detected in both group-based and individual samples, with detection rates varying by sample type (Table 2).

**Table 2:** Detection rates of swIAV-RNA in group-based and individual sample specimen as determined by RT-qPCR

sample specimen		swIAV-positive samples		swIAV-positive farms	
group samples	oral fluids	9.2%	(5/54)	16.7%	(3/18)
individual samples	nasal swab I	6.3%	(34/540)	27.8%	(5/18)
	nasal swab II	6.3%	(34/540)	16.7%	(3/18)
	bronchial swab	9.7%	(52/534)	22.2%	(4/18)
	lung sample	10.9%	(58/534)	16.7%	(3/18)

At the herd level, swIAV-RNA was detected in 7 of 18 farms (38.9%) through individual sampling, with three of these farms additionally identified as positive by group sampling. Ct-values from SH obtained OFs ranged from 26.74 to 34.90 (median: 28.31; IQR:4.77). At the individual level, 13.7% (74/540) of pigs tested positive for swIAV-RNA in at least one sample. Detailed sample-level detection patterns and corresponding Ct-values for individual samples are provided in Table 3 (see end of manuscript).

**Table 3:** Sample-level detection patterns of swIAV-RNA positive animals, including corresponding Ct-values and, where available, subtypes

farm	animal no.	Ct-values / Subtype					
		NS I	NS II	BS			LT
5	7	**31.07**	-	-			-
	8	**-**	-	25.53			-
	9	22.45 / HA-1 C.2.1	22.84 / HA-1 C.2.1	20.35 / HA-1 C.2.1			25.4 / HA-1 C.2.1
	10	29.83 / failed	39.6	28.34 / failed			32.43 / HxN1av
	11	-	-	-			31.71
	12	-	-	-			32.25
	14	-	-	-			33
	15	-	-	-			34.13
	19	-	-	-			34.79
	20	-	-	-			34.99
	21	-	-	32.82			34.57
	24	-	-	-			33.93
7	1	31.45 / failed	29.98	19.6 / HA-1B.1			34.15
	2	31.78	33.95	29.69			-
	3	26.83 / HxN2	22.5 / HA-1B.1	15.15 / HA-1B.1			24.28 / HA-1B.1
	4	29.03	29.87	18.16			24.53
	5	24.44 / HA-1B.1	23.3 / HA-1B.1	17.41 / HA-1B.1			24.3 / HA-1B.1
	6	29.97	32.14	19.78			25.65
	7	22.56 / HA-1B.1	24.46 / HA-1B.1	18.09 / HA-1B.1			27.09 / HA-1B.1
	8	30.43 / HxN2	33.13	17.95 / HA-1B.1			26.82 / HA-1B.1
	9	28.72 / HxN2	21.79 / HA-1B.1	14.6 / HA-1B.1			24.39 / HA-1B.1
	10	32.33	28.36	22.5			26.13
	11	29.86	30.12	24.54			27.16
	12	30.37	29.67	24.52			26.24
	13	25.85	26.3	18.24			22.78
	14	33.06	30.88	18.09			27.15
	15	33.38	33.36	25.68			28.5
	16	34.65	32.01	21.5			25.66
	17	31.88	24.24	17.89			29.6
	18	-	28.27	23.32			29.8
	19	-	29.49	15.32			24.55
	20	30.36	30.01	17.98			21.34
	21	32.39	31.88	17.92			25.16
	22	-	31.35	18.98			25.51
	23	-	32.79	20.22			24.72
	24	-	30.1	22.13			25.28
	25	33.62	22.24	15.59			23.78
	26	-	29.52		17.26 / HA-1B.1	21.62 / HA-1B.1	
	27	-	31.15		20.45	22.4 / HA-1B.1	
	28	-	31.22		15.34 / HA-1B.1	25.19	
	29	37.61	34.36		25.13	24.62	
	30	-	30.16		19.83	25.78	
11	20	-	-		32.27 / failed	-	
12	8	26.8 / HA-1 C.2.4	-		-	-	
	9	32.86	-		-	-	
	10	33.73	-		-	-	
	24	-	35.37		-	-	
	29	-	31.13 / failed		-	-	
15	5	-	-		27.51	26.03	
	6	-	-		26.21	27.14	
	7	-	-		26.45 / HA-1B.1	28.7 / HxN2	
	8	-	-		28.95 / HA-1B.1	28.89 / HxN2	
	9	-	-		25.75	28.56	
	10	-	-		30.91	28.66	
	11	-	-		27 / HA-1B.1	26.26 / HA-1B.1	
	12	-	-		28.12 / HA-1B.1	28.01 / HA-1B.1	
	13	-	-		28.95	29.83	
	14	-	-		28.78	29.09	
	15	-	-		30.75	29.63	
	16	-	-		27.97	30.28	
	24	-	-		29.73	29.15	
	25	-	-		30.5	28.36	
	26	-	-		30.95	28.37	
	27	-	-		30.33	29.25	
	28	-	-		-	30.02	
	29	-	-		31.08	31.06	
	30	-	-		-	29.09	
16	18	34.97	-				
	19	32.97	-		-	-	
	20	33.37	-		-	-	
	22	31.83 / failed	-		-	-	
	23	35.78	-		-	-	
	24	34.45	-		-	-	
21	15	37.57	-		-	-	

Legend: OF: oral fluids, NS: nasal swab; BS: bronchial swab; LT: lung tissue. Cells marked with (–) indicate that no swIAV RNA was detected. Empty cells represent samples that were not collected as LT and BS were unavailable for sampling at the slaughterhouse due to severe pleuritis

### Comparison of detection rates between sample materials at slaughter

Using Cochran’s Q test, significant differences in swIAV-RNA detection rates between individual sampling materials were observed (Table 2). Post-hoc pairwise comparisons showed significantly lower detection rates in upper respiratory tract samples (NS I, NS II) compared with lower respiratory tract samples (BS and LT) (NS I vs. BS: *p* < 0.001; NS I vs. LT: *p* = 0.005, Bonferroni-corrected). No statistically significant differences were observed between NS I and NS II or between BS and LT.

### Probability of swIAV detection at slaughter

The probability of detecting swIAV-RNA or antibodies in each sampling material was assessed using multivariable logistic regression (Table 4). The odds of detecting antibodies in serum samples were significantly higher (*p* < 0.001) than the probability of detecting swIAV-RNA. Samples from the lower respiratory tract (BS and LT) showed significantly higher odds of swIAV-RNA detection compared with nasal swabs (*p* < 0.05). No statistically significant differences were observed between NS I and NS II or between BS and LT.

**Table 4:** Odds ratios for swIAV detection by sample material based on multivariable logistic regression

sample specimen	reference	OR with 95% CI	*p*-value
NS II	NS I	1 (0.36; 2.77)	> 0.999
BS		2.76 (1.08; 7.07)	**0.027**
LT		3.52 (1.39; 8.90)	**0.002**
serum		1786 (626; 5097)	**< 0.001**
BS	NS II	2.76 (1.08; 7.07)	**0.027**
LT		3.52 (1.39; 8.90)	**0.002**
serum		1786 (626; 5097)	**< 0.001**
LT	BS	1.28 (0.59; 2.78)	0.911
serum		648 (267; 1570)	**< 0.001**
serum	LT	507 (216; 1188))	**< 0.001**

Legend: NS: nasal swab; BS: bronchial swab; LT: lung tissue. P-values in bold indicate significance

Agreement between sampling materials for swIAV-RNA detection ranged from fair to moderate. Fair agreement was observed between NS I and NS II (κ = 0.41; *p* < 0.001) and between NS II and BS (κ = 0.42; *p* < 0.001), while moderate agreement was found between BS and LT (κ = 0.58; *p* < 0.001). Detailed agreement estimates are presented in Table 5.

**Table 5:** Agreement between individual sampling materials for swIAV-RNA detection assessed by Cohen’s kappa

sample specimen		NS II	BS	LT
NS I	*Kappa (κ)*	**0.41**	-0.01	-0.2
	*p-value*	**< 0.001**	0.535	0.964
NS II	*Kappa (κ)*		**0.42**	0.22
	*p-value*		**< 0.001**	0.021
BS	*Kappa (κ)*			**0.58**
	*p-value*			**< 0.001**

Legend: NS: nasal swab; BS: bronchial swab; LT: lung tissue. P-values in bold indicate significance

### Quantitative detection of swIAV-RNA at slaughter

**Fig. 2:**
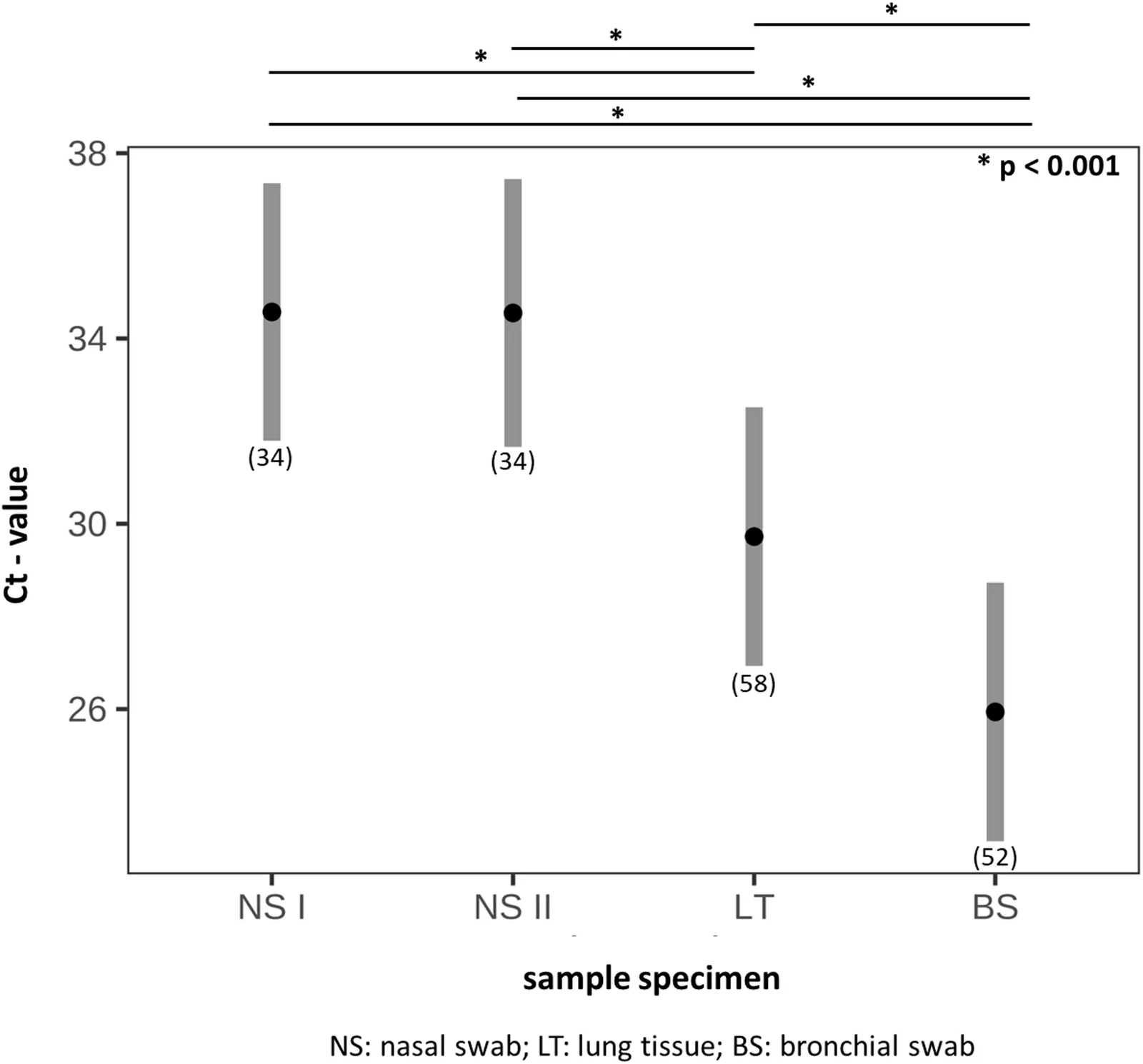
Ct-values obtained by RT-qPCR across different sample specimens at slaughter. Legend: Model-derived estimates with corresponding error bars. Black circles represent estimated marginal means from robust linear mixed-effects model (accounting for repeated measurements within individual farms). Vertical lines indicate 95% confidence interval

Regarding group-based samples, Ct-values in OFs ranged from 26.74 to 34.90 (median: 28.31; var: 12.24). On the individual level, Ct-values differed significantly between sample materials (Fig. 2). Samples from the upper respiratory tract (NS I and NS II) showed significantly higher Ct-values than samples from the lower respiratory tract (BS and LT). Among LT samples that were swIAV-RNA positive without corresponding detection in BS (*n* = 9), Ct-values were significantly higher than in LT samples with concurrent BS positivity (*p* < 0.001), indicating lower viral RNA load.

### Probability of swIAV subtyping at slaughter

To assess the suitability of different sample materials for downstream subtyping, the probability of obtaining Ct-values < 33 was evaluated using multivariable logistic regression (Table 6). Samples derived from the lower respiratory tract showed a significantly higher probability of yielding Ct-values below this threshold than samples from the upper respiratory tract. Both BS and LT samples were significantly more likely to produce Ct-values < 33 compared with nasal swabs (*p* < 0.001). No statistically significant differences were observed between NS I and NS II or between BS and LT.

**Table 6:** Odds ratios for obtaining Ct-values < 33 by sample material, indicative of suitability for downstream subtyping

sample specimen	reference	OR with 95% CI	*p*-value
NS II	NS I	1.64 (0.52; 5.17)	0.680
BS		11.0 (3.38; 35.8)	**< 0.001**
LT		10.3 (3.18; 33.6)	**< 0.001**
BS	NS II	6.69 (2.21; 20.2)	**< 0.001**
LT		6.29 (2.08; 19.0)	**< 0.001**
BS	LT	1.06 (0.44; 2.59)	0.998

Agreement between sampling materials for the detection of swIAV-RNA with Ct-values < 33 ranged from fair to substantial. Fair agreement was observed between NS I and NS II (κ = 0.38; *p* < 0.001), between NS II and BS (κ = 0.37; *p* < 0.001), and between NS II and LT (κ = 0.34; *p* < 0.001). In contrast, substantial agreement was found between bronchial swabs and lung tissue samples (κ = 0.72; *p* < 0.001). Detailed agreement estimates are provided in Table 7.

**Table 7:** Agreement between sample materials for swIAV subtyping based on Ct-values < 33 (Cohen’s kappa)

sample specimen		NS II	BS	LT
NS I	*Kappa (κ)*	**0.38**	0.10	0.02
	*p-value*	**0.001**	0.169	0.433
NS II	*Kappa (κ)*		**0.37**	**0.34**
	*p-value*		**< 0.001**	**< 0.001**
BS	*Kappa (κ)*			**0.72**
	*p-value*			**< 0.001**

Of all samples with a Ct value < 33, 46 were selected for subsequent subtyping. Of these, 32 samples were successfully subtyped, enabling subtype identification in four of six RT-qPCR–positive farms. Six samples could only be subtyped partially. The subtypes detected in this study were HA-1 C.2.1 (H1avN1EA), HA-1 C.2.4 (H1avN2G), and HA-1B.1 (H1huN2G). Subtyping success differed between sample materials, with the highest percentage of successfully subtyped samples obtained from BS (Table 8).

**Table 8:** Results of subtyping from selected swIAV positive samples with Ct-values < 33

sample specimen	positive samples (Ct < 33)	successfully subtyped samples	successfully subtyped farms	detected subtypes
NS I	23	4/10 (40%)	3	HA-1 C.2.1 (H1avN1EA), HA-1 C.2.4 (H1avN2G)
NS II	28	5/6 (83%)	2	HA-1B.1 (H1huN2G)
BS	52	13/15 (86%)	3	HA-1 C.2.1 (H1avN1EA), HA-1B.1 (H1huN2G)
LT	51	10/13 (77%)	3	HA-1 C.2.1 (H1avN1EA), HA-1B.1 (H1huN2G)
OF	4	1/2 (50%)	1	HA-1B.1 (H1huN2G)

## Discussion

Slaughterhouses offer a practical framework for pathogen monitoring, as large numbers of animals from multiple herds can be sampled efficiently at a single location, thereby reducing time and costs [40, 41]. Sampling at slaughter reduces the overall burden on farmers, as no additional animal handling or intervention during the production phase is required, and it also minimizes stress for the pigs by avoiding live‑animal restraint. In addition, access to postmortem tissues enables diagnostic approaches that may improve detection compared to sampling from living animals. Finally, sampling at slaughter allows diagnostic results to be linked to routine carcass and organ findings. If applied routinely, slaughterhouse‑based sampling could also serve as an effective early detection tool for circulating pathogens. Accordingly, the present study evaluated whether slaughterhouse sampling can support or partially substitute on-farm monitoring for swIAV detection and subtyping.

In the present study, swIAV circulation was characterized by high seroprevalence but comparatively limited viral RNA detection. Although 18 of 21 farms showed serological evidence of exposure, active infection as determined by RT-qPCR was detected in only a subset of herds during fattening and at slaughter. This discrepancy highlights the fundamental difference between cumulative exposure and active infection and reflects the narrow temporal window of viral shedding. Importantly, seropositivity at slaughter indicates prior swIAV exposure but does not allow conclusions on whether infection occurred during fattening or already in earlier production stages, which may also contribute to the discrepancy between seropositivity and limited viral RNA-detection. Since no serum samples were collected at the start of the study, this could not be ruled out, thus representing a limitation of the study. In twelve herds, all tested animals were seropositive, underlining the utility of slaughterhouse-based serology for retrospective monitoring in non-vaccinated herds. Especially in herds with enzootic infection patterns, swIAV infections often remain subclinical, hampering targeted animal selection for RT-PCR based detection of swIAV [42]. Serological assays offer a broad temporal window for detection [43], in contrast to direct detection methods, which are limited to a short period of virus shedding of around 7 days [22, 44]. Nevertheless, information on circulating swIAV subtypes is essential for epidemiological interpretation and risk assessment [22]. However, subtype identification using hemagglutination inhibition assays is resource-intensive and complicated by vaccine-induced antibodies, regular updates of test antigens, and cross-reactivity between subtypes [45–47].

Despite longitudinal sampling at three predefined time points during fattening, as well as event-based TBS sampling in herds showing respiratory distress, and sampling along the slaughter process, swIAV-RNA was detected in only 7 of the 18 seropositive farms during fattening and likewise in 7 of 18 farms at slaughter. During fattening, RT‑qPCR positivity occurred most frequently at the beginning of the fattening period, whereas fewer herds were positive at mid and late stages, and repeated detection across consecutive sampling points was uncommon. This indicates that even repeated, fixed‑interval sampling may miss short shedding events in enzootically infected herds. Importantly, targeted sampling of clinically affected pigs in fattening did not increase detection probability: despite timely TBS sampling after the onset of coughing or sneezing, swIAV-RNA was detected in only 4 of the 15 affected farms. In agreement with these findings, OF samples in this study showed comparatively high Ct‑values, reflecting low levels of viral replication indicative of predominantly subclinical infections. In contrast, previous studies report a correlation between decreasing Ct‑values in oral fluids and increasing coughing scores during acute swIAV infection [48]. Notably, agreement between the two sampling locations (on-farm vs. SH) was incomplete: of the seven farms that tested PCR‑positive on‑farm, only five were also positive at slaughter, whereas two were detected exclusively on‑farm. Conversely, two further farms were only positive at slaughter. Rather than indicating inconsistency, this partial overlap is consistent with short and intermittent viral shedding and suggests that on-farm and slaughterhouse sampling capture different temporal stages of swIAV infection dynamics.

Concerning slaughterhouse-obtained samples, swIAV detection probability strongly depended on the sample material analyzed. At the herd level, nasal swabs collected immediately after stunning identified the highest number of positive farms (5/18), supporting their role as gold standard for swIAV detection in live pigs. Interestingly, oral fluid sampling at the slaughterhouse detected fewer positive herds than individual sampling, contrasting with previous on-farm studies [18, 30]. Reduced interaction with cotton ropes in lairage pens, potentially related to transport-associated stress, likely limited the utility of this approach. The low diagnostic performance of oral fluids observed in this study likely reflects slaughterhouse-specific conditions rather than an inherent limitation of the sampling matrix. In two herds, swIAV-RNA positivity was restricted to nasal swabs collected immediately after stunning, while all subsequent samples remained negative. Positivity restricted to nasal swabs must be interpreted cautiously, as swIAV is predominantly transmitted via the airborne route and uptake during transport or in lairage pens cannot be excluded [49, 50]. Indirect transmission via contaminated environment can also not be ruled out as swIAV-RNA has been detected in dust and enrichment material [18, 30, 51]. Slaughterhouse-based RT-qPCR results may therefore reflect both farm-level infection dynamics and short-term exposure during transport or lairage, representing a limitation when interpreting individual positive findings from the upper respiratory tract.

At the individual animal level, swIAV-RNA was detected in 13.7% of pigs (74/540) sampled, which is consistent with the reported intra-herd prevalence of 11.4% in endemically infected fattening herds [52]. Samples from the lower respiratory tract outperformed upper respiratory tract samples with respect to both detection probability and viral RNA load. Detection rates and odds of positivity were significantly higher for bronchial swabs and lung tissue than for nasal swabs, consistent with the predominant replication of swIAV in the bronchial epithelium [53].

Although the same number of animals tested positive in nasal swabs before and after scalding, agreement between these sampling points was only fair. This likely reflects differences in sampling conditions: nasal swabs collected immediately after stunning were obtained under time constraints and were occasionally contaminated with blood, which may impair RNA detection [54]. In contrast, post-scalding sampling allowed more thorough collection, although washout of viral material by scalding water cannot be excluded. The absence of increased detection after scalding suggests that cross-contamination via scalding water, as described for bacterial pathogens, especially Enterobacteriaceae [55–58], is considered unlikely.

Agreement between BS and LT samples was moderate and lower than previously reported in clinically affected pigs [34]. This discrepancy may reflect differences in infection stage as swIAV infection progresses from small bronchi to terminal bronchioles and alveoli [59]. Given this infection pattern, LT samples - which predominantly contain peripheral lung structures including small bronchi, bronchioles and alveolar tissue - may capture different stages of infection than BS, which specifically target larger airways. Additional factors such as limited sample size, subtype-specific detection differences, or technical variation during tissue sampling may also have contributed.

Across all swIAV–positive samples, Ct-values were consistently lower in samples from the lower respiratory tract, indicating higher viral RNA loads. Bronchial swabs yielded the lowest Ct-values, likely reflecting broader sampling area of the primary localization of swIAV replication in the bronchial epithelium [53]. Given that multiple swIAV subtypes may co-circulate within a single herd and that pan-PCR detection alone is therefore insufficient for epidemiological interpretation, these findings are particularly relevant for downstream molecular subtyping. Previous studies have demonstrated successful subtyping at Ct-values ≤ 33 [38], a threshold met by all swIAV-positive bronchial swabs in the present study.

## Conclusion

Taken together, the results are consistent with previous findings from earlier production stages, suggesting that RT-qPCR–based swIAV monitoring in fattening pigs is likewise limited by short and intermittent viral shedding and the poor correlation between respiratory signs and detectable virus. Accordingly, longitudinal on-farm sampling with only three sampling points and slaughterhouse-based RT-qPCR investigations identified swIAV only on a subset of serologically positive herds. In contrast, serology at slaughter reliably reflected prior exposure in this non-vaccinated population; however, no conclusions can be drawn regarding when exposure occurred. Among PCR-based individual sample materials at slaughter, bronchial swabs demonstrated the most favorable overall diagnostic performance. While detection rates were comparable to those of lung tissue, bronchial swabs yielded the lowest Ct values and the highest success in downstream molecular subtyping. Slaughterhouse-based diagnostics therefore represent a valuable complementary surveillance component, particularly for serological herd-level exposure assessment and molecular characterization of circulating strains. However, they do not replace structured on-farm investigations when a detailed assessment of herd-level infection dynamics is required.

## Data Availability

The dataset used and analyzed during the current study is available from the corresponding author on reasonable request.

## Declarations

### AI-assisted copy-editing statement

Following the publisher’s guidelines, ChatGPT was used only for copy editing (grammar, spelling, and style). No original content was generated, and the authors retain full responsibility for the manuscript.
